# The effect of serum triglyceride concentration on the outcome of acute pancreatitis: systematic review and meta-analysis

**DOI:** 10.1038/s41598-018-32337-x

**Published:** 2018-09-20

**Authors:** Lóránd Kiss, Gabriella Fűr, Péter Mátrai, Péter Hegyi, Emese Ivány, Irina Mihaela Cazacu, Imre Szabó, Tamás Habon, Hussain Alizadeh, Zoltán Gyöngyi, Éva Vigh, Bálint Erőss, Adrienn Erős, Máté Ottoffy, László Czakó, Zoltán Rakonczay

**Affiliations:** 1 Department of Pathophysiology, University of Szeged, Szeged, Hungary; 2 Institute for Translational Medicine and Szentagothai Research Center, Medical School, University of Pécs, Pécs, Hungary; 3 First Department of Medicine, University of Szeged, Szeged, Hungary; 4 Department of Gastroenterology, First Department of Medicine, Medical School, University of Pécs, Pécs, Hungary; 5 Department of Cardiology and Angiology, First Department of Medicine and Szentagothai Research Center, Medical School, University of Pécs, Pécs, Hungary; 6 Department of Haematology, First Department of Medicine, Medical School, University of Pécs, Pécs, Hungary; 7 Department of Public Health Medicine, Medical School, University of Pécs, Pécs, Hungary; 8 Department of Radiology, Medical School, University of Pécs, Pécs, Hungary; 9 MTASZTE Translational Gastroenterology Research Group, University of Szeged, Szeged, Hungary

## Abstract

Elevated serum triglyceride concentration (seTG, >1.7 mM or >150 mg/dL) or in other words hypertriglyceridemia (HTG) is common in the populations of developed countries. This condition is accompanied by an increased risk for various diseases, such as acute pancreatitis (AP). It has been proposed that HTG could also worsen the course of AP. Therefore, in this meta-analysis, we aimed to compare the effects of various seTGs on the severity, mortality, local and systemic complications of AP, and on intensive care unit admission. 16 eligible studies, including 11,965 patients were retrieved from PubMed and Embase. The results showed that HTG significantly elevated the odds ratio (OR = 1.72) for severe AP when compared to patients with normal seTG (<1.7 mM). Furthermore, a significantly higher occurrence of pancreatic necrosis, persistent organ failure and renal failure was observed in groups with HTG. The rates of complications and mortality for AP were significantly increased in patients with seTG >5.6 mM or >11.3 mM versus <5.6 mM or <11.3 mM, respectively. We conclude that the presence of HTG worsens the course and outcome of AP, but we found no significant difference in AP severity based on the extent of HTG.

## Introduction

Triglyceride is a component of lipoproteins, and its reference range in the blood serum is below 1.7 mM (150 mg/dL). Nowadays, a high-fat diet and resultant obesity are common; therefore, the prevalence of elevated serum triglyceride concentration (seTG) is high, affecting approximately 27% of all adults^1^. In the disease of hypertriglyceridemia (HTG), the fasting seTG exceeds 1.7 mM. The aetiology of HTG may be primary (caused by genetic mutations), but it is most commonly secondary^2^. The causes of secondary HTG include obesity, unhealthy diet or lifestyle (such as heavy alcohol consumption), pregnancy, hypothyroidism, hepatic steatosis, nephrotic syndrome, type-2 diabetes mellitus, and some drugs (e.g. glucocorticoids, oestrogen and tamoxifen)^2,3^. The extent of HTG has been classified by the Endocrine Society into the following groups based on fasting seTG: mild (1.7 to 2.3 mM), moderate (2.3 to 11.2 mM), severe (11.3 to 22.4 mM) and very severe HTG (>22.4 mM)^4^. However, the classification of HTG based on fasting seTG was somewhat different according to the National Cholesterol Education Program Adult Treatment Panel III guideline^5^. They specified borderline-high seTG from 1.7 to 2.2 mM, high seTG between 2.2 and 5.6 mM, and very high seTG above 5.6 mM. HTG is associated with different comorbidities. In the general population, people with moderate or severe non-fasting seTG compared with individuals with non-fasting seTG in the normal range have a significantly higher risk for ischemic heart disease, ischemic stroke and mortality^1^. HTG is also an important risk factor for the development of acute pancreatitis (AP)^6^.

AP is a sudden inflammation of the pancreas that lasts for a short period (from days to some weeks)^6^. The severity of the disease can be classified into three groups based on the modified Atlanta criteria: mild, moderately severe and severe^7^. Moderately severe and severe forms of AP are associated with various local and systemic complications which also influence the rate of mortality. Necrosis, infections, abscesses and/or pseudocysts can occur in the pancreas. AP may also cause systemic complications, such as sepsis, systemic inflammatory response syndrome, and transient or persistent organ failure^7–9^. Organ failure most commonly affects the lungs, kidneys and cardiovascular system. About 60–80% of AP cases are due to massive alcohol consumption and gallstone disease, whereas 1–9% of the cases are HTG-induced^2,6,10^. In pregnancy, up to 56% of AP cases are HTG-related^2^. It is widely accepted that severe HTG markedly increases the risk for AP^2,11^. However, some authors define HTG-induced AP (HTG-AP) when seTG is >5.6 mM^10^. There is no significant evidence for HTG-AP at <5.6 mM seTG^12^. Beyond the increased risk for AP in severe HTG, previous publications have indicated that there is a relationship between seTG and the severity of AP^10,13,14^, even in the case of mild or moderate HTG^15–18^. Nawaz *et al*.^15^ reported that HTG independently and proportionally correlates with persistent organ failure regardless of AP aetiology. Zeng *et al*.^16^ showed that seTG >2.26 mM increases the risk for systemic and local complications in acute biliary pancreatitis. However, some studies have shown no relationship between seTG and the severity of AP^19,20^. To clarify this discrepancy, earlier efforts have aimed to analyse the effect of seTG on the severity of AP through meta-analyses^10,13,14^. Only one meta-analysis has been published in which the authors investigated the effect of seTG on the outcomes of AP^13^. However, the groups of patients classified by seTG overlapped, which is a significant limitation of that study.

To the best of our knowledge, there is no meta-analysis where the effect of several different and well-defined seTGs have been investigated on the course of AP. Therefore, our aim was to evaluate and compare the effects of normal seTG with the effect of mild, moderate and severe HTG on the severity, mortality and other complications of AP. In addition, various ranges within the HTG were compared.

## Methods

This systematic review and meta-analysis was conducted according to the protocol previously registered in the PROSPERO database (https://www.crd.york.ac.uk/PROSPERO/, ID: CRD42017071264). The methodology for this analysis followed recommendations by Stroup *et al*.^21^ and the guidelines for the Preferred Reporting Items for Systematic Reviews and Meta-Analysis Protocols (Supplementary Table S1)^22^. The analysis was based on the Problem, Intervention, Comparison intervention, and Outcome (PICO) model^22^. The problem was AP. The intervention was HTG with various groups formed for the analysis: >1.7, 1.7–5.6, 1.7–11.3, >5.6 and >11.3 mM seTG. The comparison interventions were normal (<1.7), <5.6, 1.7–5.6, 1.7–11.3 and <11.3 mM seTG. Different outcomes were investigated: AP severity, mortality, pancreatic necrosis, persistent organ failure (POF) and multi-organ failure (MOF), pulmonary and renal failure, and admission to an intensive care unit (ICU).

### Article Search Strategy

The search was carried out in late August 2017. Observational prospective and retrospective cohorts, and case control studies were identified in Embase (published from 1948 to July 2017) and PubMed Library (published from 1961 to July 2017). Furthermore, ClinicalTrials.gov was also screened for additional unpublished data. The search contained the following terms for Embase: pancreatitis AND (‘triglyceride’/exp OR triglyceride OR hypertriglyceridemia OR ‘hyperlipidemia’/exp OR ‘hyperlipidemia’) AND [english]/lim AND (‘human’/de OR patient OR patients) NOT (‘conference abstract’/it OR ‘review’/it OR ‘case report’/de OR ‘nonhuman’/de OR ‘practice guideline’/de). The following terms were used for PubMed: pancreatitis AND (hyperlipidaemia OR hyperlipidemia OR triglycerides OR triglyceride OR hypertriglyceridaemia OR hypertriglyceridemia) AND (human OR patient OR patients) AND English NOT “case reports”[Publication Type]. The search terms for Clinicaltrials.gov were pancreatitis and hypertriglyceridemia.

### Eligibility criteria

Articles were included if they fulfilled the following criteria:

1. Case control or cohort studies.
2. Studies involving AP patients.
3. HTG (>1.7 mM) was present in at least one of the groups under investigation
4. SeTGs were defined.
5. Outcome data were provided for at least one of the following: severity of AP according to the revised Atlanta Classification, mortality, pancreatic necrosis, POF, MOF, pulmonary failure, renal failure and intensive care unit (ICU) admission
6. Written in English.

The seTG in different groups used as controls was below 1.7, 5.6 or 11.3 mM as well as within the 1.7–5.6 and 1.7–11.3 mM ranges. Systematic reviews, meta-analyses, reviews, conference abstracts, letters, replies, reports, commentaries, notes, case studies, animal studies, practice guidelines and non-English-language (e.g. Chinese-, Polish- and German-language) articles were excluded from the analysis.

### Data extraction and quality assessment of the articles included

Relevant studies were manually screened by two independent researchers (LK and GF). The investigators read and selected the articles to be included in the statistical analysis and extracted the data. The Newcastle–Ottawa Scale (NOS) was used (Supplementary Table S2) to assess the quality of the articles included^23,24^. Since seTG decreases rapidly when food intake is restricted^25^, the NOS was supplemented with another scoring system in which the articles were also evaluated based on the timing of the seTG measurement (Supplementary Table S2).

### Statistical analysis

The statistical analysis was performed with Stata 11 SE (StataCorp LLC, College Station, TX, USA). The numbers of patients with regard to AP severity, mortality, pancreatic necrosis, POF, MOF, pulmonary failure, renal failure and ICU admission were used to calculate odds ratios (OR) to compare these outcomes in different seTG groups. ORs were pooled using the random effects model with the DerSimonien–Laird estimation and displayed on forest plots. Summary OR estimation, p value and 95% Confidence Interval (CI) were calculated. ORs with corresponding CIs and p values are indicated in the text according to the following order: OR [CI; p]. P < 0.05 was considered a significant difference from summary OR = 1. The different ORs were compared using the analysis of variance with random effect weights.

#### Heterogeneity and publication bias

Statistical heterogeneity was analysed using the I^2^ statistic and the chi-square test to acquire probability values; p < 0.05 was defined to indicate significant heterogeneity. The small-study effect was visually investigated on funnel plots.

## Results

### Study selection

**Figure 1:**
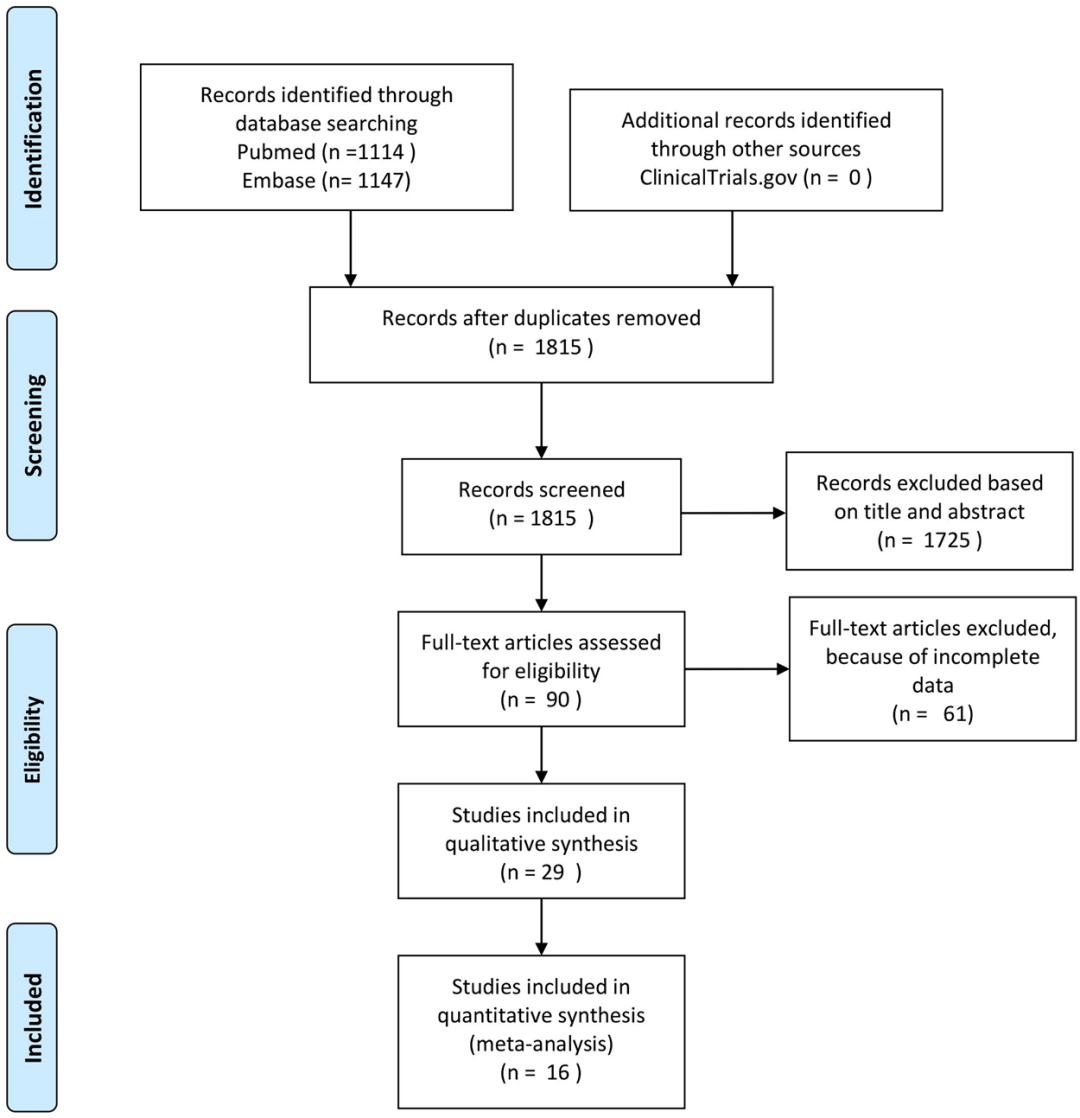
Flow diagram for article selection.

The search for articles in three databases resulted in 2261 records (Fig. 1). After removing duplicates and screening titles and abstracts, 90 articles were assessed in full text for eligibility. Of these manuscripts, 29 prospective and retrospective cohorts seemed to be suitable for data collection. However, in 13 publications, seTGs were defined inappropriately (e.g.<1.88 mM was identified as normal) or the outcome data could not be used. Therefore, these 13 publications were removed from the assessment, and only 16 articles were included in the statistical analysis (in which the seTG ranges or the outcome data were appropriate). These studies were published between January 2000 and March 2016.

### Characteristics of studies

Both single- (13) and multicentre (3) cohort studies were included. Population sizes ranged from 43 to 3203, and six trials involved over 300 patients. The aetiology of AP was noted in all the studies, eight comprised HTG-AP (>11.3 mM seTG), and twelve contained alcoholic and biliary pancreatitis patients. Other aetiologies, such as post-ERCP, idiopathic, mixed and drug-induced AP, were also included in some articles. The studies were performed in the following countries: China (nine cohorts), the USA (three cohorts), Hungary (two cohorts), the UK (one cohort) and Spain (one cohort). Table 1 summarizes the characteristics of the cohorts involved. During the quality assessment, we evaluated patient selection, comparability of the groups, outcome data, and the timing of the seTG measurement. These quality scores are depicted in the corresponding figures.

**Table 1:** Characteristics of the studies included in the meta-analysis.

First author, year	Source	Country	Study design	Inclusion period	Centre	Patient number: used for analysis/total (n/n)	AP aetiology	AP severity classification system	Groups based on seTG (mM); patients (n)	AP outcomes
Jiang, 2005^54^	Chin J Dig Dis	CHN	NA	Jan 2000–Jan 2002	S	99/99	B, A, O	N.R.	<1.7; 71 >1.7; 28	# Hospital stays # Ranson and APACHE II scores # Pulmonary, cardiovascular and renal failure # Pancreatic pseudocyst and infected necrosis
Balachandra, 2006^20^	Int J Clin Pract	UK	P	2001	S	40/43	B, A, I, pE	N.R.	<1.8; 26 >1.8; 14	# Pancreatic pseudocyst or necrosis # Respiratory insufficiency # Atlanta mild and severe cases
Deng, 2008^55^	World J Gastroenterol	CHN	R	Mar 2003–Dec 2004	S	176/176	A, B, D, L-asparaginase chemotherapypregnancy	N.R.	<5.65; 131 ≥5.65; 45	# Ranson, APACHE II and Balthazar’s CT scores # Pulmonary (ARDS) and renal failure # Acute hepatitis # Shock # Encephalopathy # Infection # Mortality
Baranyai, 2012^56^	Clin Lipidol	HUN	R	Jan 2007–Dec 2009	S	351/351	HTG, O	N.R.	<11.3; 328 >11.3; 23	# Complications # Severe AP # Sepsis # Necrosis # Mortality # Hospital stays
Ivanova, 2012^57^	Hepatobiliary Pancreat Dis Int	ESP	P	Mar 2006–Feb 2007	S	133/133	B, A, I, HTG, O	N.R.	<11.28; 126 >11.28; 7	# ICU admission # Mortality # Hospital stays
Zeng, 2014^16^	Am J Med Sci	CHN	R	N.D.	S	340/340	B, B + HTG	Revised Atlanta Classification	<1.70; 250 1.70–2.25; 18 2.26–5.64; 31 ≥ 5.65; 41	# Mild, moderately severe and severe AP # Ranson score # Pulmonary, cardiovascular and renal failure # Pancreatic pseudocyst and infected necrosis # Sepsis # SIRS # Pancreatic necrosis, pseudocyst and abscess
Nawaz, 2015^15^	Am J Gastroenterol	USA	P	Jun 2003–Jun 2004	S	201/201	B, A, I, HTG, O	Revised Atlanta Classification	<1.70; 115 1.70–2.26; 20 2.27–11.32; 41 ≥11.33; 25	# Persistent organ failure # ICU admission # Mortality # Pancreatic necrosis
Zheng, 2015^58^	Pancreas	CHN	R	Jan 2006–Dec 2010	M	2461/2461	B, A, HTG, O	N.R.	<11.3; 2206 >11.3; 255	# Mild and severe AP cases # Mortality
Chen, 2016^42^	Pancreatology	CHN	NA	Mar 2015–Mar 2016	S	57/57	B, A, I, HL	Revised Atlanta Classification	<11.33; 30 >11.33; 27	# Mild, moderately severe and severe AP cases # Pancreatic necrosis # Organ failure, multiple organ dysfunction syndrome # Infected necrosis # Mortality # Length of hospitalization # Length of ICU stay # APACHE II, SOFA and BISAP scores
Goyal, 2016^43^	North Am J Med Sci	USA	R	Jan 2009–Jun 2015	S	177/177	HTG, A	Revised Atlanta Classification	<11.33; 147 >11.33; 30	# Mild, moderately severe and severe AP cases # BISAP, SIRS scores and Balthazar index # Hospital stays, ICU admission # Surgical intervention # Mortality
Párniczky, 2016^*,^^44^	PLoS One	HUN	P	Jan 2013–Jan 2015	M	113/600	B, I, A, A + D, HL, pE, O	Revised Atlanta Classification	<1.7; 59 1.7–5.64; 28 5.65–11.33; 4 ≥11.33; 22	# Mild, moderately severe and severe AP cases # Mortality
Tai, 2016^45^	Gastroenterol Res Pract	CHN	R	Feb 2010–Jan 2014	S	294/294	B, HTG	Revised Atlanta Classification	<11.33; 168 >11.33; 126	# Mild, moderately severe and severe AP cases # Mortality # Pulmonary failure (ALI/ARDS) # Renal insufficiency # Cardiovascular insufficiency # Gastrointestinal bleeding # Sepsis # Multiple organ disfunction syndrome # Hospital stays
Sue, 2017^17^	Pancreas	USA	R	2006–2013	M	2519/2519	B, A, O	Revised Atlanta Classification	<1.7; 1729 1.7–2.26; 251 2.27–5.66; 308 5.67–11.32; 82 ≥11.33; 149	# No organ failure # Transient organ failure # Multiple or persistent organ failure
Wan, 2017^18^	Lipids Health Dis	CHN	R	Jan 2005–Dec 2013	S	1539/1539	B, A, HTG, I, O	Revised Atlanta Classification	<1.7; 1078 1.7–2.23; 107 2.23–11.2; 242 >11.2; 112	# Mild, moderately severe and severe AP cases # Pancreatic necrosis # Organ failure, persistent or multiple organ failure # Persistent SIRS # Mortality # Length of hospitalization # Length of ICU stay # Acute peripancreatic fluid collection # Acute necrotic collection
Wu, 2017^46^	Pancreatology	CHN	R	Jul 2009–Jul 2014	S	262/262	B, A, O	Revised Atlanta Classification	<1.7; 104 1.7–5.67; 72 5.67–11.33; 47 >11.33; 39	# Pulmonary failure (ARDS) # Renal failure (acute kidney injury) # Shock # Infected pancreatic necrosis # Mortality # Hospital stays # ICU stays
Zhu, 2017^47^	Pancreas	CHN	NA	Jan 2005–Dec 2012	S	3203/3260	B, I, HL, A, O, (Mixed)	Revised Atlanta Classification	<11.33; 2736 >11.33; 467	# Mild, moderately severe and severe AP cases # Mortality

Abbreviations: A, alcoholic; ALI, acute lung injury; AP, acute pancreatitis; APACHE II, acute physiology and chronic health evaluation; ARDS, acute respiratory distress syndrome; B, biliary; BISAP, bedside index for severity in acute pancreatitis; CHN, China; CT, computed tomography; D, overeating and/or high-fat diet; ESP, Spain; HTG, hypertriglyceridaemic; HUN, Hungary; I, idiopathic; ICU, intensive care unit; M, multicentre; NA, not available; NR, not relevant, because the data from AP severity were not used for the meta-analysis; O, others; P, prospective; pE, post- endoscopic retrograde cholangiopancreatography; R, retrospective; S, single-centre; SIRS, Systemic inflammatory response syndrome; SOFA, sequential organ failure assessment; seTG, serum triglyceride concentration; UK, United Kingdom; USA, United States of America. *The authors had access to the raw data in Párniczky et al. (2016)^44^ because of the overlap between the authors. Therefore, it was possible to create new groups based on seTG which were not presented in the original publication (Supplementary Table S3).

### Clinical outcomes

#### Comparing the effects of HTG vs. normal seTG on the severity of AP

**Figure 2:**
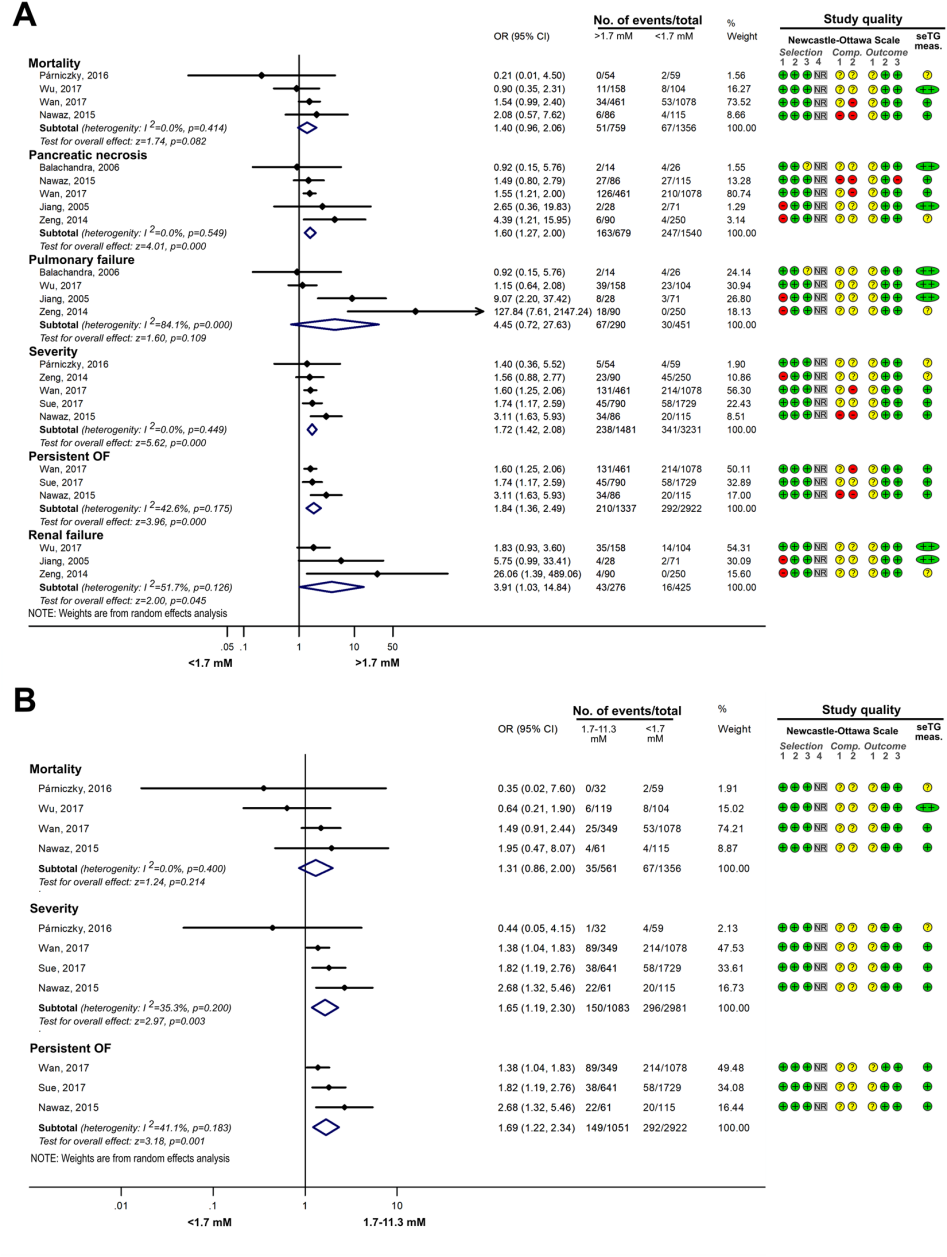
The effects of seTG >1.7 and 1.7–11.3 mM vs. <1.7 mM on AP severity, mortality, pancreatic necrosis, pulmonary and renal failure, and persistent OF. (**A**) Forest plot shows the influence of seTG over 1.7 mM compared with normal seTG (<1.7 mM). (**B**) The outcomes for the 1.7–11.3 mM seTG group were compared with those in patients with normal seTG (<1.7 mM). Filled rhombuses represent the risk ratio derived from the studies analysed. Horizontal bars represent 95% CI. Empty rhombuses show the overall effect (OR is the middle of the rhombus and CIs are the edges). The quality of studies was assessed by the Newcastle-Ottawa Scale with the timing of the seTG measurement (for more details see Supplementary Table S2). Abbreviations: AP, acute pancreatitis; CI, confidence interval; Comp., comparability; NR, not relevant; meas., measurement; OF, organ failure; OR, odds ratio; seTG, serum triglyceride concentration.

**Figure 3:**
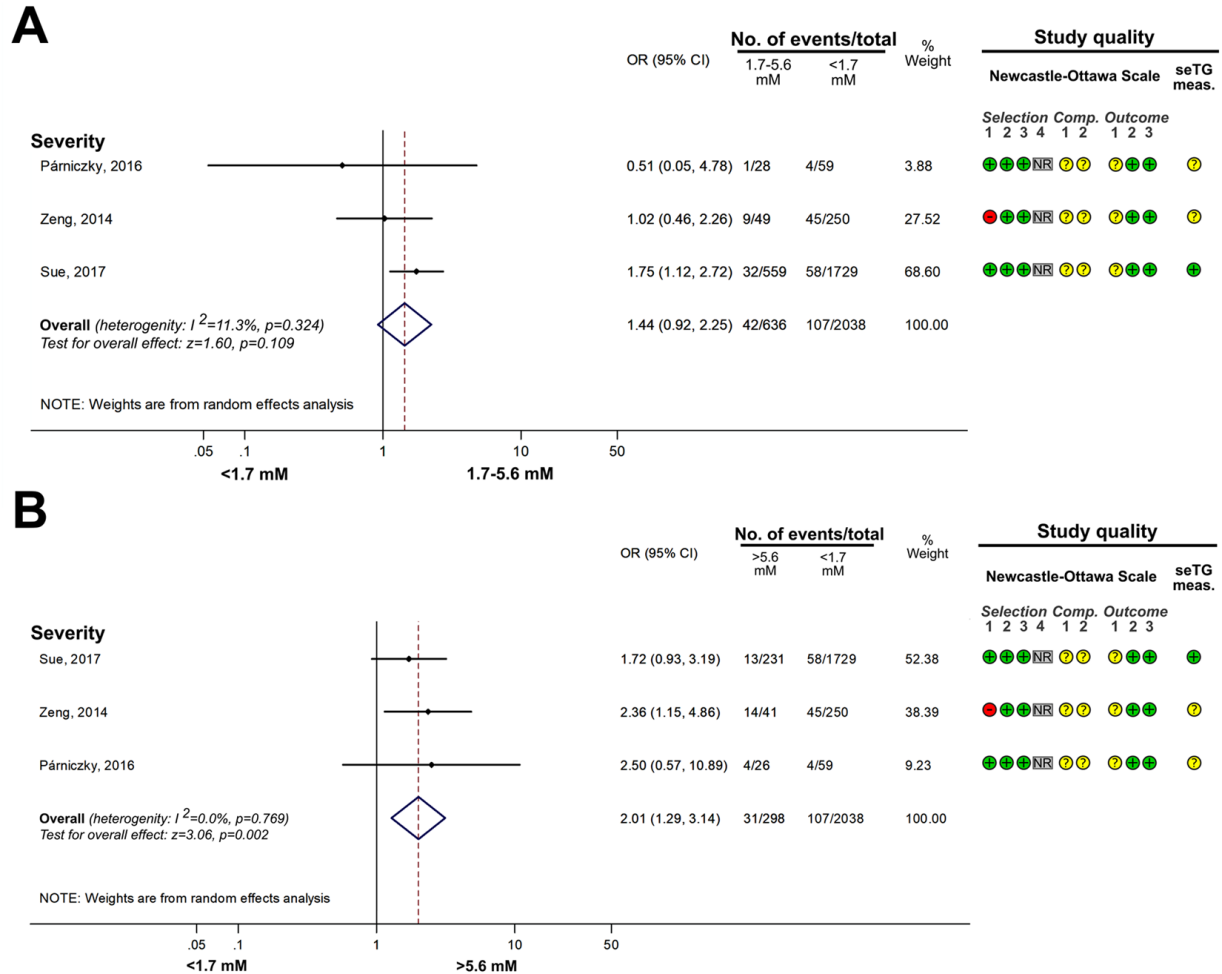
The effects of serum triglyceride concentration (seTG) at 1.7–5.6 mM and >5.6 mM vs. <1.7 mM on acute pancreatitis (AP) severity. (**A**) Forest plot shows the influence of 1.7–5.6 mM seTG compared with normal seTG (<1.7 mM). (**B**) The outcome of the >5.56mM seTG group was compared with the outcomes of patients with normal seTG (<1.7 mM). Filled rhombuses represent the risk ratio derived from the manuscripts analysed. Horizontal bars represent 95% CI. Empty rhombuses show the overall effect, odds ratio (OR) is the middle of the rhombus, and confidence intervals (CI) are the edges. The quality of studies was assessed by the Newcastle-Ottawa Scale with the timing of the seTG measurement (for more details see Supplementary Table S2). Abbreviations: CI, confidence interval; Comp., comparability; NR, not relevant; meas., measurement; OR, odds ratio; seTG, serum triglyceride concentration.

**Figure 4:**
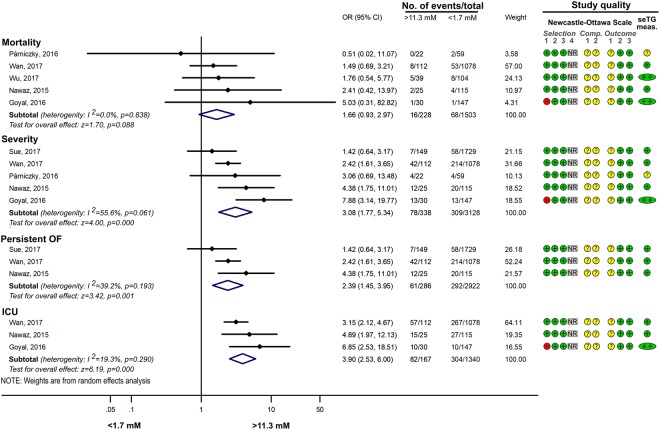
Forest plot showing the effect of seTG >11.33 mM vs. <1.7 mM on AP severity, mortality, persistent OF and the need for ICU. Filled rhombuses represent the risk ratio derived from the articles analysed. Horizontal bars represent 95% CI. Empty rhombuses show the overall effect (OR is the middle of the rhombus and CIs are the edges). The quality of studies was assessed by the Newcastle-Ottawa Scale with the timing of the seTG measurement (for more details see Supplementary Table S2). Abbreviations: AP, acute pancreatitis; CI, confidence interval; comp., comparability; ICU, intensive care unit; NR, not relevant; meas., measurement; OR, odds ratio; seTG, serum triglyceride concentration.

**Figure 5:**
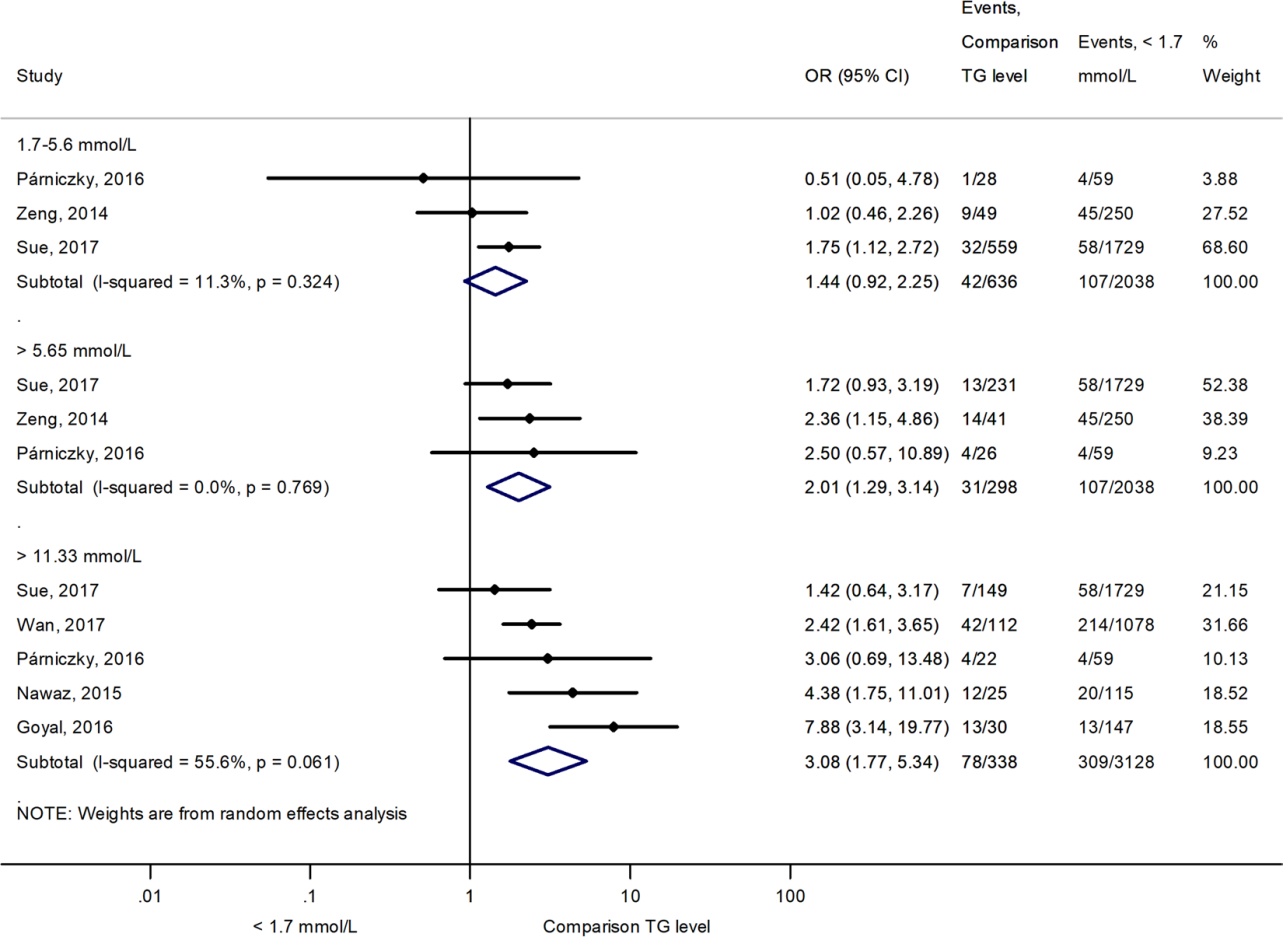
The effects of seTG of 1.7–5.6, >5.6 and >11.3 mM vs. <1.7 mM on AP severity. The difference between ORs in seTG subgroups (1.7–5.6, >5.6 and >11.3 mM) were compared. The probability value for the difference in OR was 0.108 after the analysis.

Different groups were created based on the extent of HTG, and the outcomes for AP were compared with those in the normal (<1.7 mM) seTG group. Figure 2A shows how HTG affects the course of AP. HTG significantly increased the number of severe AP cases (severity), pancreatic necrosis, persistent OF and renal failure compared to the non-HTG group (Fig. 2A). However, HTG did not significantly increase the odds for mortality and pulmonary failure compared to the <1.7 mM group. Analysing the effect of seTG in the range from 1.7 to 11.3 mM showed results similar to the previous comparison. The severity of AP and the incidence of POF significantly increased in the 1.7–11.3 mM range compared to the <1.7 mM seTG group, while it had no significant effect on the mortality of the patients (Fig. 2B). HTG was further divided into ranges of 1.7–5.6, >5.6 and >11.3 mM seTG. Figure 3A shows that the severity of AP was not significantly different in patients with 1.7–5.6 mM seTG compared to the <1.7 mM group. However, seTGs >5.6 mM significantly increased the risk for severe AP in patients with OR of 2.01 [CI: 1.29–3.14; p = 0.002] compared to seTG <1.7 mM (Fig. 3B). The presence of severe and very severe HTG (>11.3 mM) markedly increased the severity of AP (OR = 3.08 [CI: 1.77–5.34; p = 0.000]), POF (OR = 2.39 [CI: 1.45–3.95; p = 0.001]) and ICU admission (OR = 3.90 [CI: 2.53–6.00; p = 0.000]), but there was no significant elevation in mortality compared to the normal seTG group (Fig. 4). The OR values for AP severity showed an increasing trend (1.44 [CI: 0.92–2.25; p = 0.109]; 2.01 [CI: 1.29–3.14; p = 0.002]; 3.08 [CI: 1.77–5.34; p = 0.000]) in groups with HTG (1.7–5.6, >5.6 and >11.3 mM, respectively), compared to the reference group with seTG <1.7 mM (Figs 3 and 4, Table 2). Therefore, these results were compared statistically (Fig. 5). However, no significant differences (p = 0.108) were found between the three ORs.

**Table 2:** Summary of the groups compared based on seTG.

Comparison intervention	Intervention	Outcome OR [*CI*; *p*]								*Figure*
		AP Severity	Mortality	Pancreatic necrosis	Persistent OF	Multiple OF	Pulmonary failure	Renal failure	ICU admission	
**<1**.**7**	**>1**.**7**	**1**.**72 [*1***.***42–2***.***08***; ***0***.***000*]**	*1*.*40* [*0*.*96–2*.*06*; *0*.*082*]	**1**.**60 [*1***.***27–2***.***00***; ***0***.***000*]**	**1**.**84 [*1***.***36–2***.***49***; ***0***.***000*]**		*4*.*45* [*0*.*72–27*.*63*; *0*.*109*]	**3**.**91 [*1***.***03–14***.***84***; ***0***.***045*]**		2A
	**1**.**7–11**.**3**	**1**.**65 [*1***.***19–2***.***30***; ***0***.***003*]**	*1*.*31* [*0*.*86–2*.*00*; *0*.*214*]		**1**.**69 [*1***.***22–2***.***34***; ***0***.***001*]**					2B
	**1**.**7–5**.**6**	*1*.*44* [*0*.*92–2*.*25*; *0*.*109*]								3A
	**>5**.**6**	**2**.**01 [*1***.***29–3***.***14***; ***0***.***002*]**								3B
	**>11**.**3**	**3**.**08 [*1***.***77–5***.***34***; ***0***.***000*]**	*1*.*66* [*0*.*93–2*.*97*; *0*.*088*]		**2**.**39 [*1***.***45–3***.***95***; ***0***.***001*]**				**3**.**90 [2**.***53–6***.***00***; ***0***.***000*]**	4
**<5**.**6**	**>5**.**6**	**1**.**87 [*1***.***21–2***.***89***; ***0***.***005*]**	**2**.**75 [*1***.***28–5***.***92***; ***0***.***01*]**				**4**.**23 [*1***.***05–17***.***11***; ***0***.***043*]**	**3**.**74 [*2***.***14–6***.***52***; ***0***.***000*]**		6A
**1**.**7–5**.**6**	**>5**.**6**	*1*.*60* [*0*.*73–3*.*51*; *0*.*245*]								6B
**<11**.**3**	**>11**.**3**	**1**.**66 [*1***.***28–2***.***16***; ***0***.***000*]**	**1**.**64 [*1***.***11–2***.***44***; ***0***.***014*]**	**2**.**28 [*1***.***62–3***.***21***; ***0***.***000*]**		*1*.*55* [*0*.*75–3*.*22*; *0*.*236*]			**3**.**11 [2**.***20–4***.***39***; ***0***.***000*]**	7
**1**.**7–11**.**3**	**>11**.**3**	*1*.*5* [*0*.*88–2*.*54*; *0*.*132*]	*1*.*34* [*0*.*71–2*.*55*; *0*.*366*]		*1*.*41* [*0*.*87–2*.*28*; *0*.*166*]					8

Bold cells indicate significant differences between the groups (p < 0.05), Italic cells show no significant difference (p > 0.05), and empty cells stand for no comparison for that outcome. Under outcomes, the numbers in bold indicate the OR values and square brackets contain CI and p values for the respective comparisons. Abbreviations: AP, acute pancreatitis; CI, confidence interval; ICU, intensive care unit; OF, organ failure.

The small-study effect was checked by placing the data acquired from articles on funnel plots (Supplementary Figs 1–3). A visual inspection of the funnel plots revealed no apparent asymmetry; therefore, we found no signs of the small-study effect.

#### The effect of different ranges of HTG on AP

**Figure 6:**
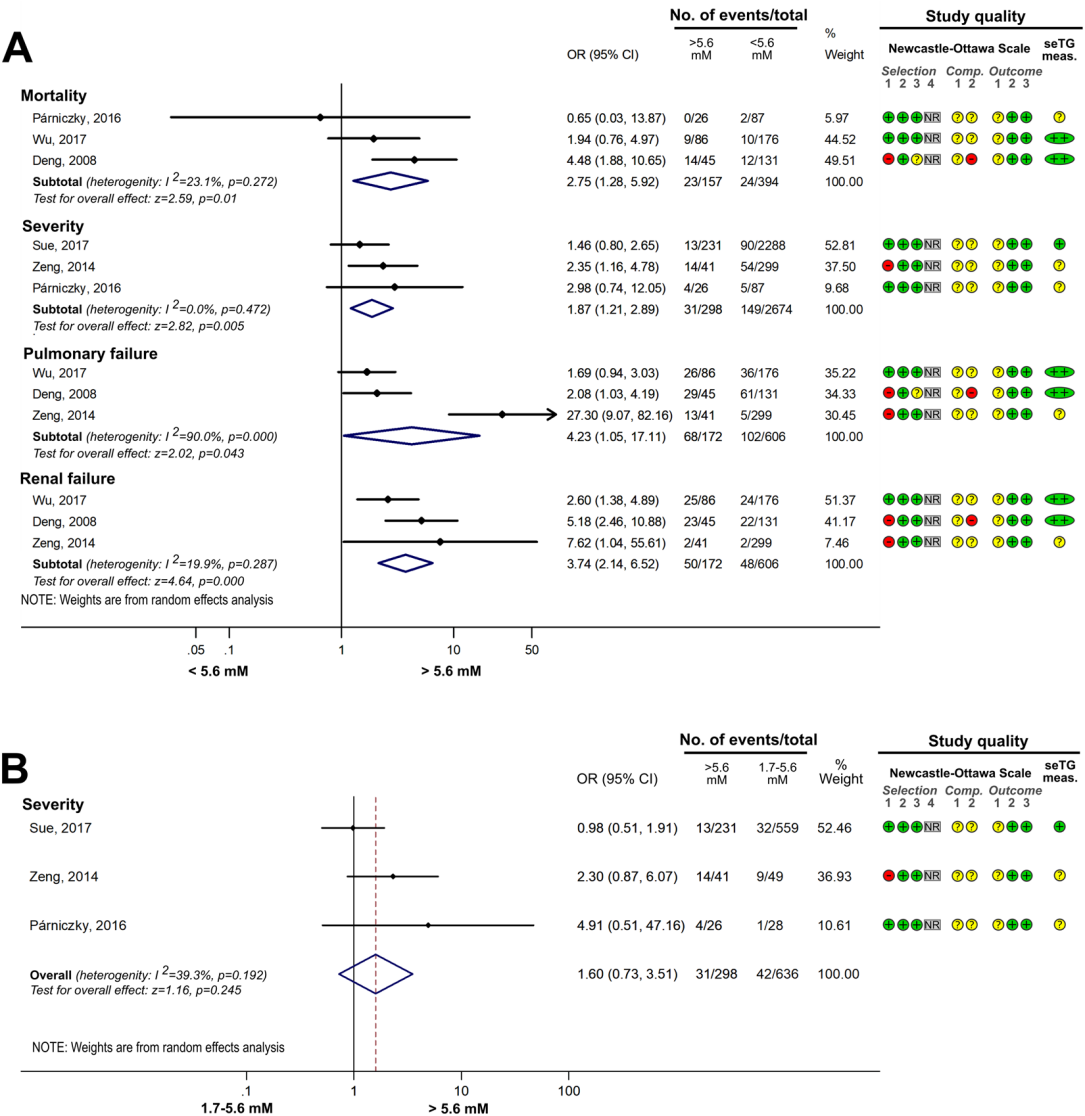
Forest plot showing the effect of seTG >5.6 mM vs. <5.6 or 1.7–5.6 mM on AP severity, mortality, and pulmonary and renal failure. (**A**) Forest plot shows the influence of seTG over 5.6 mM compared with that of seTG <5.6 mM. (**B**) The AP severity in the > 5.6 mM seTG group was compared with that in patients with seTG in the 1.7–5.6 mM range. Filled rhombuses represent the risk ratio derived from the articles analysed. Horizontal bars represent 95% CI. Empty rhombuses show the overall effect (OR is the middle of the rhombus and CIs are the edges). The quality of studies was assessed by the Newcastle-Ottawa Scale with the timing of the seTG measurement (for more details see Supplementary Table S2). Abbreviations: AP, acute pancreatitis; CI, confidence interval; comp., comparability; NR, not relevant; meas., measurement; OR, odds ratio; seTG, serum triglyceride concentration.

**Figure 7:**
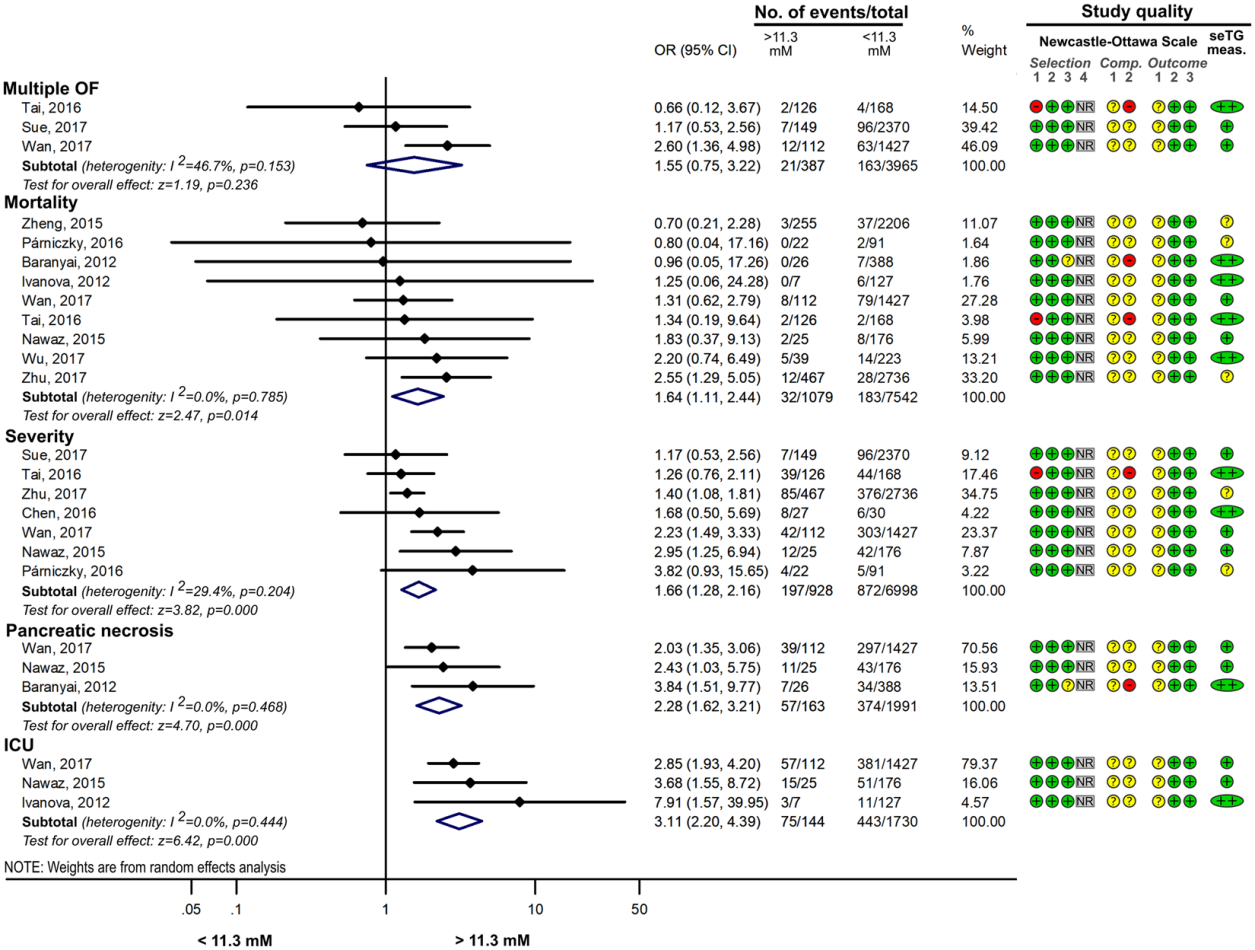
Forest plot showing the effect of seTG >11.3 mM vs. <11.3 on AP severity, mortality, pancreatic necrosis, the need for ICU admission and multiple OF. Filled rhombuses represent the risk ratio derived from the articles analysed. Horizontal bars represent 95% CI. Empty rhombuses show the overall effect (OR is the middle of the rhombus and CIs are the edges). The quality of studies was assessed by the Newcastle-Ottawa Scale with the timing of the seTG measurement (for more details see Supplementary Table S2). Abbreviations: AP, acute pancreatitis; CI, confidence interval; comp., comparability; ICU, intensive care unit; NR, not relevant; meas., measurement; OF, organ failure; OR, odds ratio; seTG, serum triglyceride concentration.

**Figure 8:**
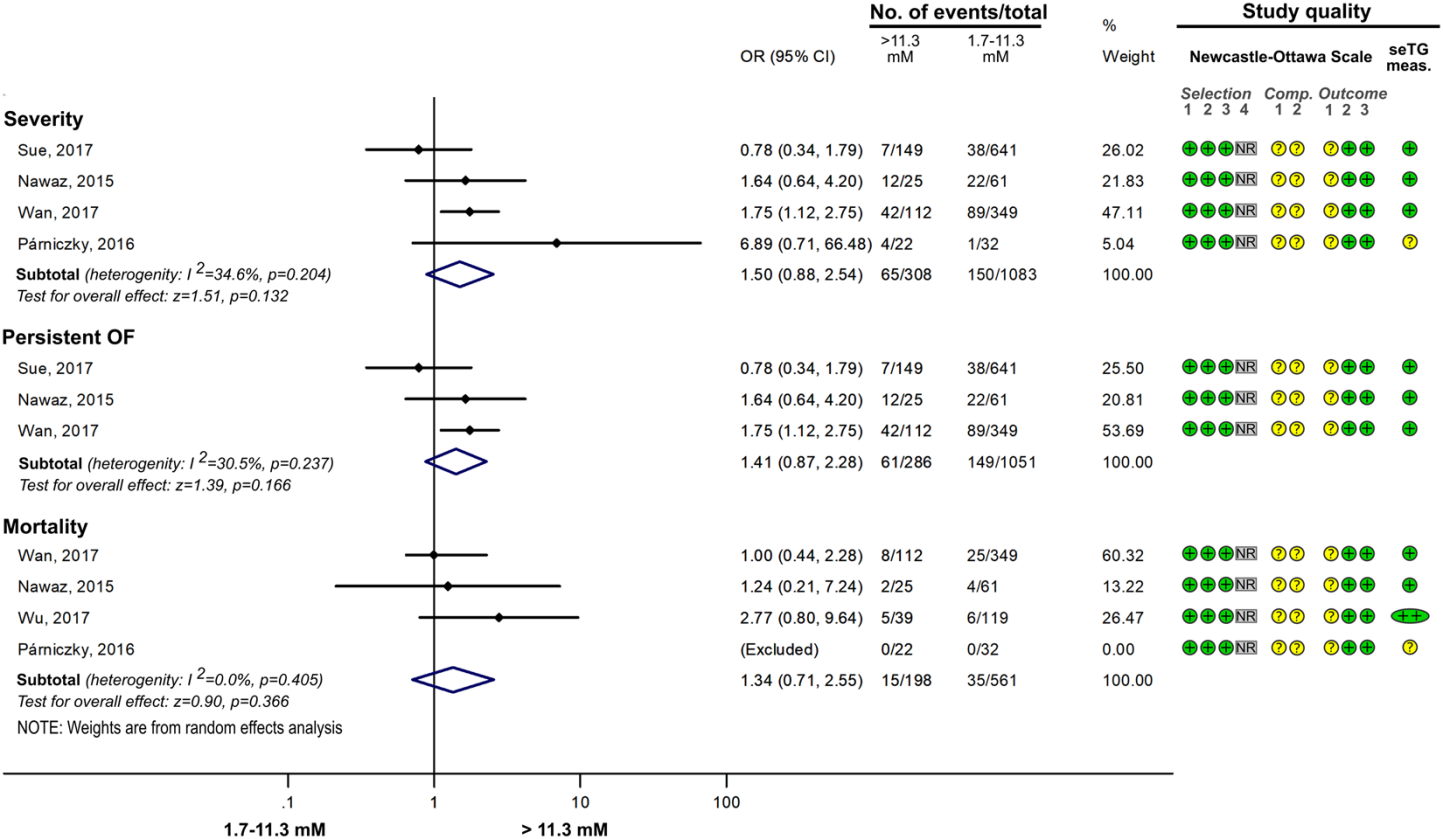
Forest plot showing the effect of seTG >11.3 mM vs. 1.7–11.3 on AP severity, mortality and persistent OF. Filled rhombuses represent the risk ratio derived from the articles analysed. Horizontal bars represent 95% CI. Empty rhombuses show the overall effect (OR is the middle of the rhombus and CIs are the edges). The quality of studies was assessed by the Newcastle-Ottawa Scale with the timing of the seTG measurement (for more details see Supplementary Table S2). Abbreviations: AP, acute pancreatitis; CI, confidence interval; comp., comparability; NR, not relevant; meas., measurement; OF, organ failure; OR, odds ratio; seTG, serum triglyceride concentration.

If seTG is elevated, the extent of the increase could also have an impact on the course of AP. Comparing the effect of seTG below and above 5.6 mM showed that seTG higher than 5.6 mM significantly increased the risk for severe AP, mortality, and pulmonary and renal failure (Fig. 6A). However, the severity of AP was not significantly different in HTG patients with seTG of 1.7–5.6 mM vs. >5.6 mM (Fig. 6B). Severe and very severe HTG (>11.3 mM seTG) significantly increased the OR of severe AP, mortality, pancreatic necrosis and ICU admission compared to group with seTG < 11.3 mM, but it did not influence the occurrence of MOF (Fig. 7). Interestingly, when the effect of severe and very severe HTG was compared with mild and moderate HTG (seTG 1.7–11.3 mM), no significant difference was revealed between the two groups with regard to AP severity, mortality and POF (Fig. 8). All the groups compared on the basis of seTG are summarized in Table 2. The data extracted from articles were depicted on funnel plots to test for the small-study effect (Supplementary Figures 4–6). Visual inspection of the funnel plots showed no evident asymmetry; therefore, we found no signs of the small-study effect.

## Discussion

In this study, we investigated the effect of different seTGs on the outcome of AP. HTG worsens the course of AP compared to the normal seTG group. However, an increase in seTG up to 5.6 mM did not significantly influence the severity of AP. Selecting subgroups within HTG (>5.6; >11.3 mM) resulted in a significant elevation of ORs (2.01 and 3.08, respectively) for severity when all the groups were compared with the effect of normal seTG on AP severity. These data suggest that HTG aggravates the course of AP. Our findings are in line with earlier animal studies, in which hyperlipidaemia increased the severity of AP^26–30^.

The underlying mechanism by which HTG exacerbates the severity of AP is unknown. One of the possible processes is that pancreatic lipases metabolize seTG to non-esterified fatty acids (NEFA)^31,32^. These NEFA are toxic and cause MOF to worsen the outcome of AP^33–36^. The administration of NEFA induced sustained elevation of [Ca^2+^] in pancreatic acinar cells and inhibited mitochondrial function and ATP production^37,38^. Consequently, NEFAs cause damage to acinar and vascular endothelial cells, thus leading to inflammation^39^. Our earlier studies have also indicated that fatty acids inhibit CFTR activity and decrease the HCO_3_^−^ and fluid secretion of pancreatic ductal cells^40,41^. In addition, in the case of HTG, the concentration of chylomicrons is elevated. This increases blood viscosity, which impairs blood flow and results in pancreatic ischemia and acidosis^31,32^. Since AP is not induced in all patients with HTG^10^, we assume that other factors also contribute to the development of AP.

Interestingly, the mortality of patients did not show statistically significant differences between groups (Figs. 2–4; p values: 0.082; 0.214; 0.088), which is likely to be the result of the small number of patients with this outcome. The odds for complications (SIRS, POF, involving pulmonary, renal, and circulatory failure) were significantly increased in groups with HTG. Although mortality is related to disease complications, the results from the statistical analysis for mortality were not in line with the outcomes for AP (e.g. severity, POF, necrosis). Therefore, further investigation of HTG-AP would be beneficial with respect to mortality.

Although there is no unanimous definition for HTG-AP, it is widely accepted that AP with seTG >11.3 mM is HTG-related^12^. However, some researchers consider HTG-AP to be defined by a seTG threshold >5.6 mM^10^. Therefore, this encouraged us to investigate the relationships between the extent of HTG and the outcome of AP (Figs. 6–8). SeTG > 5.6 mM significantly worsened the outcomes for AP when compared with the seTG < 5.6 mM group, while there was no difference when seTG > 5.6 mM was compared with seTG in the 1.7–5.6 mM range (p value: 0.245). Similar results were seen at a cut-off seTG of 11.3 mM. SeTG > 11.3 mM caused more severe AP than seTG < 11.3 mM, but when the effect of seTG > 11.3 mM was compared with that of seTG in the 1.7–11.3 mM range, no significant difference was seen between the two groups (p values: 0.132; 0.366; 0.166). These comparisons also support our previous assumption that compared to normal seTG, HTG is associated with an increased risk for severe AP and complications. However, we could not detect any significant differences in AP severity based on the degree of HTG. Further studies would be important to clarify the relationship between the extent of HTG and the severity of AP.

Most of the earlier attempts to investigate the effect of HTG on the severity of AP via meta-analysis were unsuccessful due to the small number of available observational studies^10,14^, except for Wang *et al*.^13^, which was published last year after our PROSPERO registration. It analysed 14 articles, five of which overlapped with studies included in our paper^13^. A careful look at Wang *et al*.^13^ revealed that inconsistent data and grouping abound. Two groups were defined for the comparisons, the TG-related AP (TGAP) group and the non-TG-related AP (NTGAP) group. The patients categorized as TGAP had seTG > 11.3 mM or >5.6mM with a previous history of HTG. Otherwise, patients were categorized into the NTGAP group. However, we are unsure how this categorization relates to their statistical analysis because in some of the articles involved the seTGs were classified as <2.26 or <1.7 mM and used for the NTGAP group, and the TGAP group contained patients with seTG > 2.26 or >1.7 mM. For example, Nawaz *et al*.^15^ had 115 patients with normal (<1.7 mM) seTG who were included in the NTGAP group, while the 86 patients with HTG (seTG > 1.7 mM) were listed in the TGAP group. Another example of this discrepancy could be found in Fig. 2 in their article^13^, where Zeng *et al*. (2014) involved groups with seTG < 1.7 (NTGAP) and ≥5.65 mM (TGAP), Yin *et al*. (2015) consisted of groups with seTG < 5.6 (NTGAP) and >5.6 mM (TGAP), Tariq *et al*. (2016) contained groups with seTG < 2.27 (NTGAP) and ≥2.27 mM (TGAP), and Jiang *et al*. (2005) comprised groups with seTG < 1.7 (NTGAP) and >1.7 mM (TGAP). However, all these different elevations of seTG were used within one analysis. Furthermore, some errors could also be identified for the patient numbers used for the analysis (e.g. in Fig. 2, the patients with NTGAP in Huang *et al*. (2014) should be 4/828 instead of 4/1459). Thus, Wang *et al*.^13^ compared two groups of patients, one with seTG > 1.7 mM and one with seTG < 5.65 mM, where the two groups overlap between 1.7 and 5.65 mM seTG. The authors proved that the AP group with higher seTG had an increased risk for systemic complications and an elevated mortality rate compared to patients with lower seTG. Our results partly confirm the findings from Wang *et al*.^13^.

In 2016 and 2017, eight well-written articles^17,18,42–47^ were published in which the effect of HTG on the severity of AP was investigated, and this allowed us to prepare a meta-analysis by combining those cohorts with those in earlier papers. However, our study has several limitations: (1) Although the literature is more extensive nowadays, most of our analyses contained a small number of articles (generally 3–5 studies). (2) The different populations (China or the USA) and the various baseline data (e.g. body mass index, age and sex) could represent a bias. (3) Aetiologies (biliary or alcoholic) for AP differ. (4) Only English-language articles were included in this study, which can affect the results. (5) There were no statistical significances for all the investigations even if the ORs were high (e.g. OR = 4.45 with p value of 0.109). A further increase in the number of articles and patients could clarify any discrepancies related to ORs or significance. (6) Meng *et al*.^48^ observed significant differences (more than ±10%) between the seTG values obtained using various commercially available kits. (6) Significant heterogeneities were detected for some analyses. (7) Notably, seTG changes dynamically, which relates to food intake and fasting. Current treatment protocols for most AP patients include fasting at the beginning of hospitalization, except for suspected severe AP cases where early enteral feeding (within 48 hours) is recommended^49,50^. Fasting results in a rapid (within 48 hours) drop of seTG^10^ and measuring seTG 48 or 72 hours after the admission might underrepresent levels at the onset of AP. Dominguez-Munoz *et al*.^25^ demonstrated a dramatic decrease in seTG during fasting: seTG falls from approx. 30 mM to 5 mM within three days. Other authors also confirmed this phenomenon^2,10,51^. To take this bias into account, we scored the articles based on the timing of the seTG measurement. Having high scores for NOS and seTG measurement timing represents good quality for the selection of articles for this meta-analysis.

To improve the design of further retrospective or prospective cohorts related to HTG-AP, we would suggest some recommendations. It is advisable to keep the time interval for the seTG measurement consistent. Preferably, it should be performed within 48 hours after the onset of the first symptoms and repeated regularly. Publishing the medical history of patients with respect to seTG-lowering therapies would also be advantageous, e.g. describing the regular use of statins or fibrates, which could decrease the incidence of AP^52^. If patients have HTG on admission for AP, then lipid-lowering therapy (such as plasmapheresis) is recommended according to guidelines. The use of these therapies would also improve further studies. Due to large differences between the kits used for seTG determination, standardizing the method for seTG measurement is also recommended. Based on basic discoveries, cohort analyses, clinical studies and meta-analyses, early intervention (e.g. heparin and/or insulin therapies and plasmapheresis) to normalise HTG may be beneficial for patients, and this should be investigated in randomised controlled trials.

**Figure 9:**
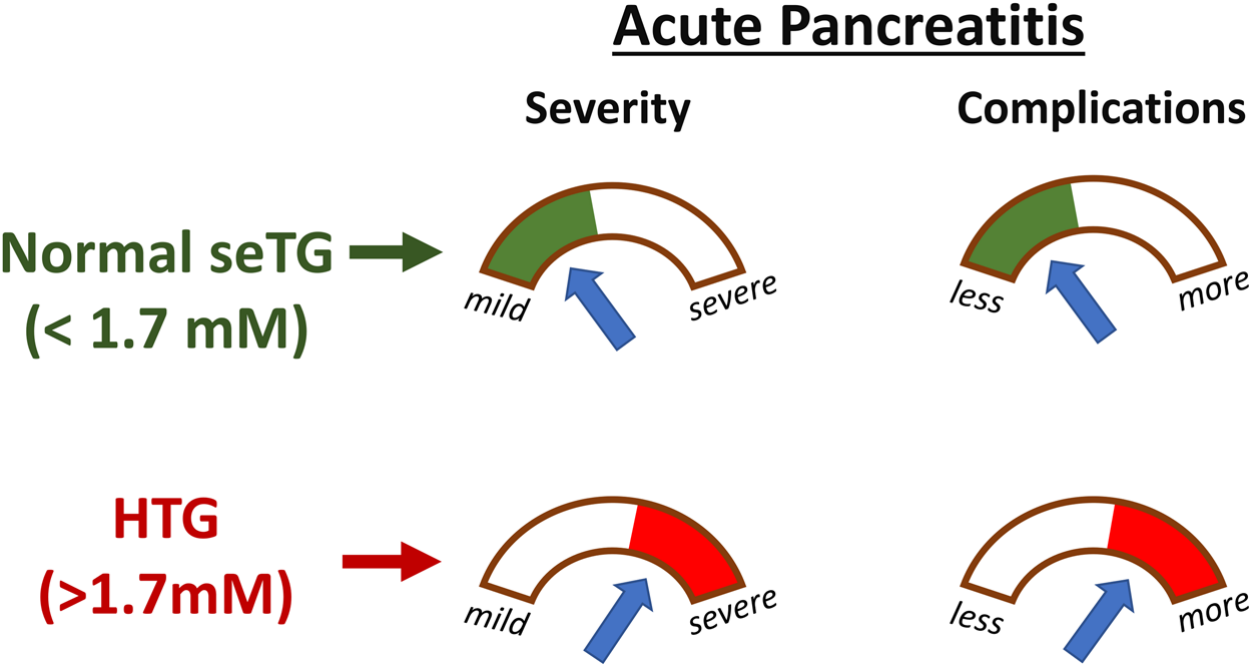
Schematic diagram shows the effect of normal seTG and HTG on the severity and complications of AP. Abbreviations: HTG, hipertriglyceridemia; seTG, serum triglyceride concentration.

In conclusion, HTG worsens the severity of AP and increases the risk for local and systemic complications (Fig. 9). However, there was no difference in AP severity based on the extent of HTG^53^.

## Electronic supplementary material

Supplementary Information
