# Supplementary material for: The effect of serum triglyceride concentration on the outcome of acute pancreatitis: systematic review and meta-analysis
Source: Sci Rep. 2018 Sep 20;8:14096. doi: 10.1038/s41598-018-32337-x (PMC6147944; doi:10.1038/s41598-018-32337-x)
Supplement: Supplementary file 1 — Supplementary Information [file 41598_2018_32337_MOESM1_ESM.pdf]

The effect of serum triglyceride concentration on the outcome of acute pancreatitis: systematic review and meta-analysis

Lóránd Kiss, Gabriella Fűr, Péter Mátrai, Péter Hegyi, Emese Ivány, Irina Mihaela Cazacu, Imre Szabó, Tamás Habon, Hussain Alizadeh, Zoltán Gyöngyi, Éva Vigh, Bálint Erőss, Adrienn Erős, Máté Ottoffy, László Czakó, Zoltán Rakonczay Jr

Supplementary Table S1. Checklist as the Transparent Reporting System of Systematic Reviews and Meta-Analyses Statement recommend.

Section/topic	#	Checklist item	Reported on page #
**TITLE**			
Title	1	Identify the report as a systematic review, meta-analysis, or both.	1
**ABSTRACT**			
Structured summary	2	Provide a structured summary including, as applicable: background; objectives; data sources; study eligibility criteria, participants, and interventions; study appraisal and synthesis methods; results; limitations; conclusions and implications of key findings; systematic review registration number.	2
**INTRODUCTION**			
Rationale	3	Describe the rationale for the review in the context of what is already known.	3-4
Objectives	4	Provide an explicit statement of questions being addressed with reference to participants, interventions, comparisons, outcomes, and study design (PICOS).	5
**METHODS**			
Protocol and registration	5	Indicate if a review protocol exists, if and where it can be accessed (e.g., Web address), and, if available, provide registration information including registration number.	5
Eligibility criteria	6	Specify study characteristics (e.g., PICOS, length of follow-up) and report characteristics (e.g., years considered, language, publication status) used as criteria for eligibility, giving rationale.	5
Information sources	7	Describe all information sources (e.g., databases with dates of coverage, contact with study authors to identify additional studies) in the search and date last searched.	7
Search	8	Present full electronic search strategy for at least one database, including any limits used, such that it could be repeated.	5
Study selection	9	State the process for selecting studies (i.e., screening, eligibility, included in systematic review, and, if applicable, included in the meta-analysis).	7
Data collection process	10	Describe method of data extraction from reports (e.g., piloted forms, independently, in duplicate) and any processes for obtaining and confirming data from investigators.	5
Data items	11	List and define all variables for which data were sought (e.g., PICOS, funding sources) and any assumptions and simplifications made.	5
Risk of bias in individual studies	12	Describe methods used for assessing risk of bias of individual studies (including specification of whether this was done at the study or outcome level), and how this information is to be used in any data synthesis.	6
Summary measures	13	State the principal summary measures (e.g., risk ratio, difference in means).	7
Synthesis of results	14	Describe the methods of handling data and combining results of studies, if done, including measures of consistency (e.g., I^2) for each meta-analysis.	6

Section/topic	#	Checklist item	Reported on page #
Risk of bias across studies	15	Specify any assessment of risk of bias that may affect the cumulative evidence (e.g., publication bias, selective reporting within studies).	6
Additional analyses	16	Describe methods of additional analyses (e.g., sensitivity or subgroup analyses, meta-regression), if done, indicating which were pre-specified.	7
**RESULTS**			
Study selection	17	Give numbers of studies screened, assessed for eligibility, and included in the review, with reasons for exclusions at each stage, ideally with a flow diagram.	7
Study characteristics	18	For each study, present characteristics for which data were extracted (e.g., study size, PICOS, follow-up period) and provide the citations.	7
Risk of bias within studies	19	Present data on risk of bias of each study and, if available, any outcome level assessment (see item 12).	7-8
Results of individual studies	20	For all outcomes considered (benefits or harms), present, for each study: (a) simple summary data for each intervention group (b) effect estimates and confidence intervals, ideally with a forest plot.	7-8
Synthesis of results	21	Present results of each meta-analysis done, including confidence intervals and measures of consistency.	7-8
Risk of bias across studies	22	Present results of any assessment of risk of bias across studies (see Item 15).	7-8
Additional analysis	23	Give results of additional analyses, if done (e.g., sensitivity or subgroup analyses, meta-regression [see Item 16]).	7
**DISCUSSION**			
Summary of evidence	24	Summarize the main findings including the strength of evidence for each main outcome; consider their relevance to key groups (e.g., healthcare providers, users, and policy makers).	9
Limitations	25	Discuss limitations at study and outcome level (e.g., risk of bias), and at review-level (e.g., incomplete retrieval of identified research, reporting bias).	9-10
Conclusions	26	Provide a general interpretation of the results in the context of other evidence, and implications for future research.	10
**FUNDING**			
Funding	27	Describe sources of funding for the systematic review and other support (e.g., supply of data); role of funders for the systematic review.	10

Supplementary Table S2. The quality of the articles in our meta-analysis was assessed by the Newcastle–Ottawa Scale (NOS) with an additional criterion including the timing of the serum triglyceride concentration (seTG) measurement. The following table lists the investigated questions.

	**Newcastle-Ottawa Scale**	**High-quality items carrying a low risk of bias** (+)	**Low-quality items carrying:**<br>• **high risk of bias** (-)<br>• **unknown risk of bias** (?)
**Selection**	**Item 1:** Representativeness of the initial study population	Non-selected groups of AP patients. The main etiologies (alcohol, biliary, HTG induced AP) are presented in the article. There is no unnecessary exclusion during the patient involvement (+)	Selected group(s) of AP patients (eg. only biliary and HTG induced AP) (-)
	**Item 2:** Selection of the non-exposed cohort	The control and the compared (HTG) groups were selected from the same population of patients (+)	The control and the compared (HTG) groups were selected from different population of patients (-)
	**Item 3:** Ascertainment of exposure	The measured data was recorded. (+)	No description about the data recording (?)
	**Item 4:** Demonstration that outcome of interest was not present at start of study.	This is not relevant in case of our study	
**Comparability**	**Item 1:** Comparability of cohorts on the basis of the design or analysis:<br>Study controls for body mass index	The groups with various seTG ranges do not differ significantly in case of the body mass index (+)	The groups with various seTG ranges significantly differ in case of the body mass index (-)<br><br>There is no statistical test for the corresponding groups with various seTG ranges in case of the body mass index (?)
	**Item 2:** Comparability of cohorts on the basis of the design or analysis:<br>Study controls for age	The groups with various seTG ranges do not differ significantly in case of the age (+)	The groups with various seTG ranges significantly differ in case of the age (-)<br><br>There is no statistical test for the corresponding groups with various seTG ranges in case of the age (?)
**Outcome**	**Item 1:** Assessment of outcome	The investigation of the AP outcome was blinded to seTGs on follow-up (+)	The investigation of the AP outcome was unblinded to seTGs on follow-up (-)<br><br>No description of blinded fashion of the outcome assessment (?)
	**Item 2:** Was follow-up long enough for outcomes to occur?	Outcomes (e.g. mortality, organ failure, AP severity assessment) were assessed during the hospital stay (+)	-
	**Item 3:** Adequacy of follow-up	Complete follow-up or subjects lost to follow up unlikely to introduce bias: description provided of those lost (+)	-

**Timing of the seTG measurement**		SeTGs were measured on admission or within 24h ++<br><br>SeTGs were measured within 72h +	Timing of seTG measurement was not stated ?

Abbreviation: AP, acute pancreatitis; HTG, hypertriglyceridemia; seTG, serum triglyceride concentration.

Supplementary Table S3. Raw data from Parniczky et. al. (2016). The boxes contain the numbers of patients in the different serum triglyceride concentration (seTG) groups. Abbreviations: AP, acute pancreatitis; mM, mmol/L.

seTG (mM)	Patients	Mild AP	Moderate AP	Severe AP	Mortality
<1.7	59	33	22	4	2
1.7–5.64	28	22	5	1	0
5.65–11.33	4	2	2	0	0
≥11.33	22	7	11	4	0
**Total**	**113**	**64**	**40**	**9**	**2**

Supplementary Figures

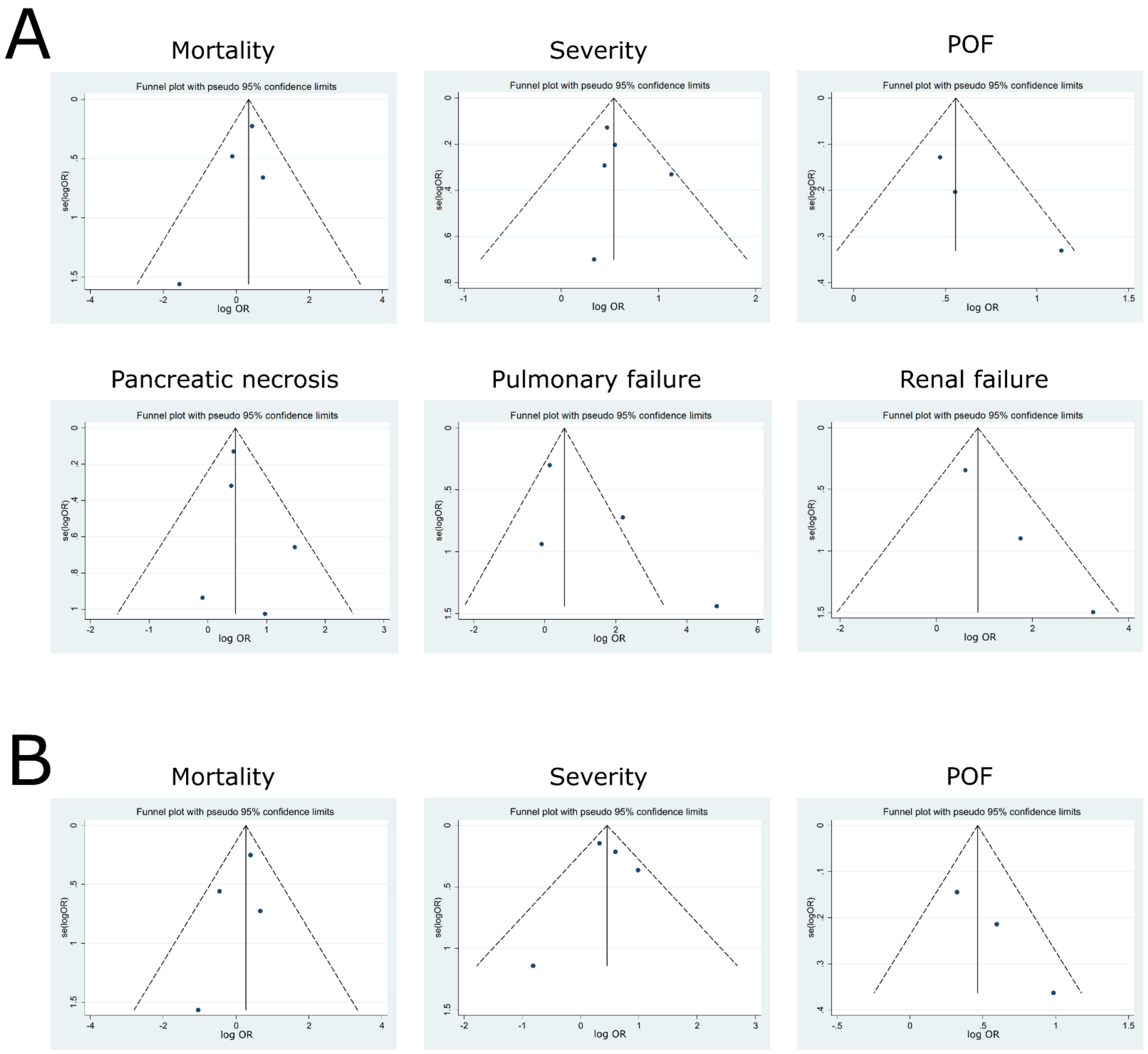

Supplementary Figure 1. Forest plots of mortality, severity, persistent organ failure (POF), pancreatic necrosis, and pulmonary and renal failure related to the meta-analysis where the >1.7 (A) and 1.7–11.3 mM (B) seTG vs. <1.7 mM seTG groups were compared (Fig. 2A and 2B, respectively). The two oblique lines mark the pseudo-95% confidence limits.

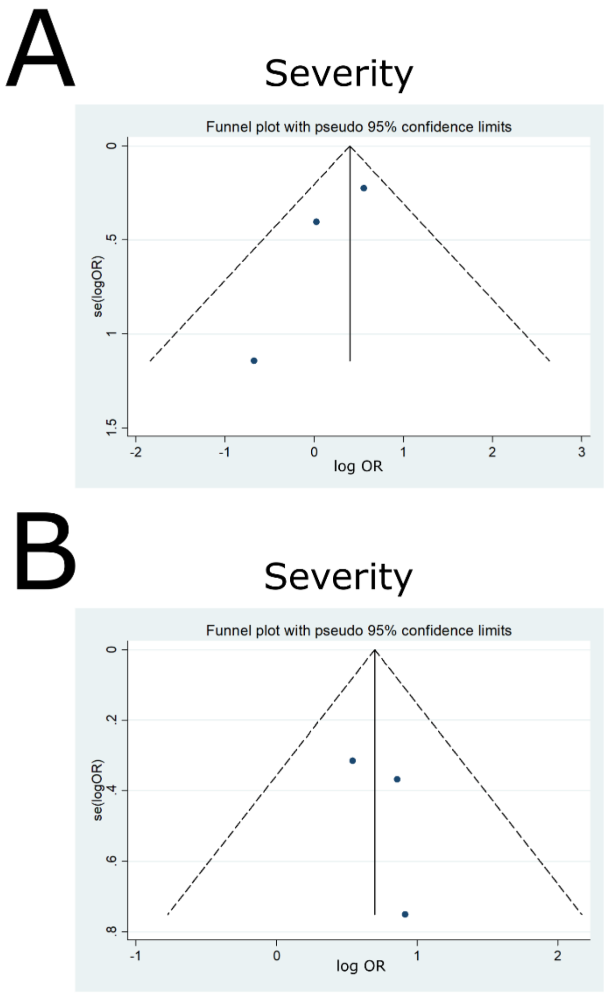

Supplementary Figure 2. Forest plots of severity related to the meta-analysis where the 1.7–5.6 (A) and >5.6 mM (B) seTG vs. <1.7 mM seTG groups were compared (Fig. 3A and 3B, respectively). The two oblique lines mark the pseudo-95% confidence limits.

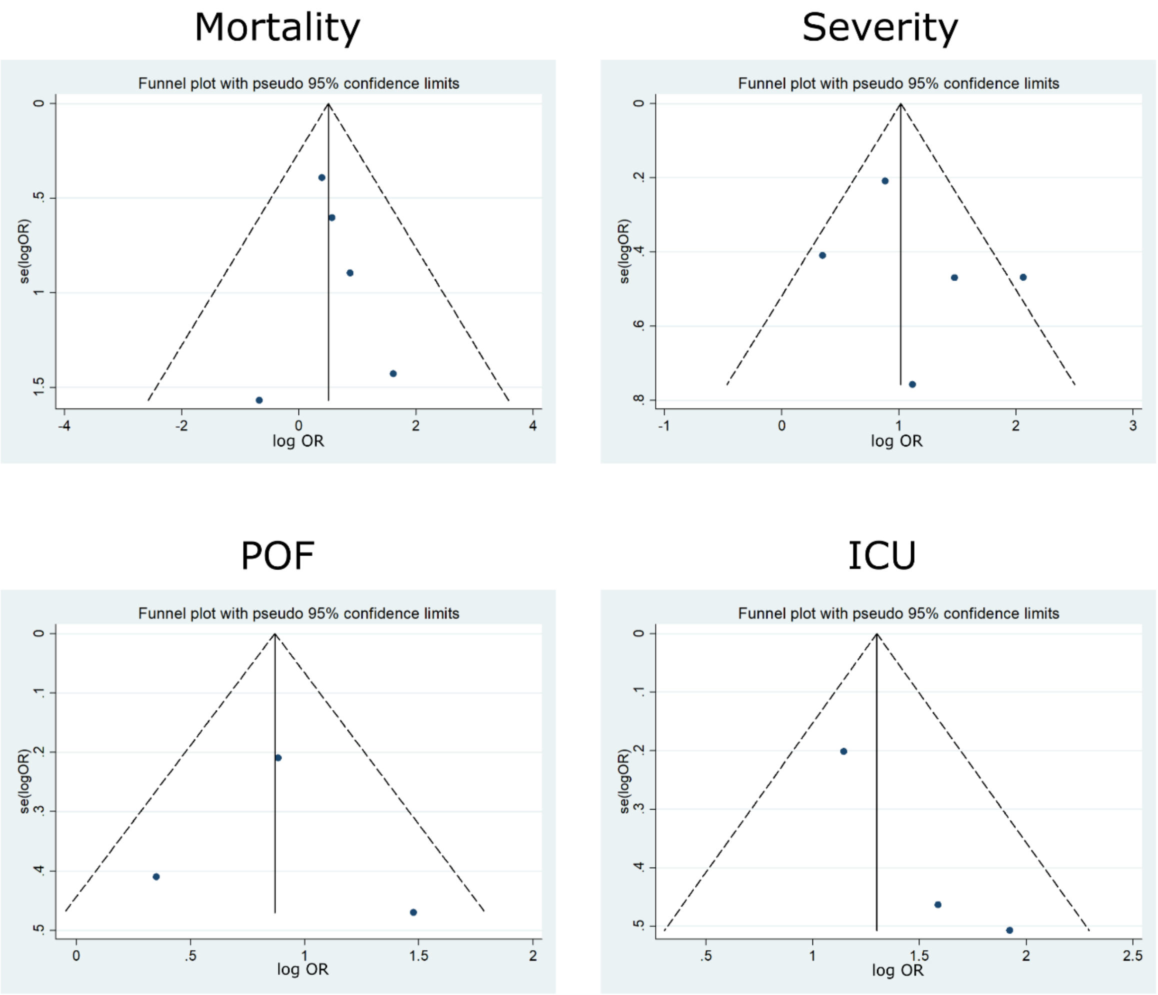

Supplementary Figure 3. Forest plots of mortality, severity, persistent organ failure (POF) and intensive care unit admission (ICU) related to the meta-analysis where the >11.3 mM vs. <1.7 mM seTG groups were compared (Fig. 4). The two oblique lines mark the pseudo-95% confidence limits.

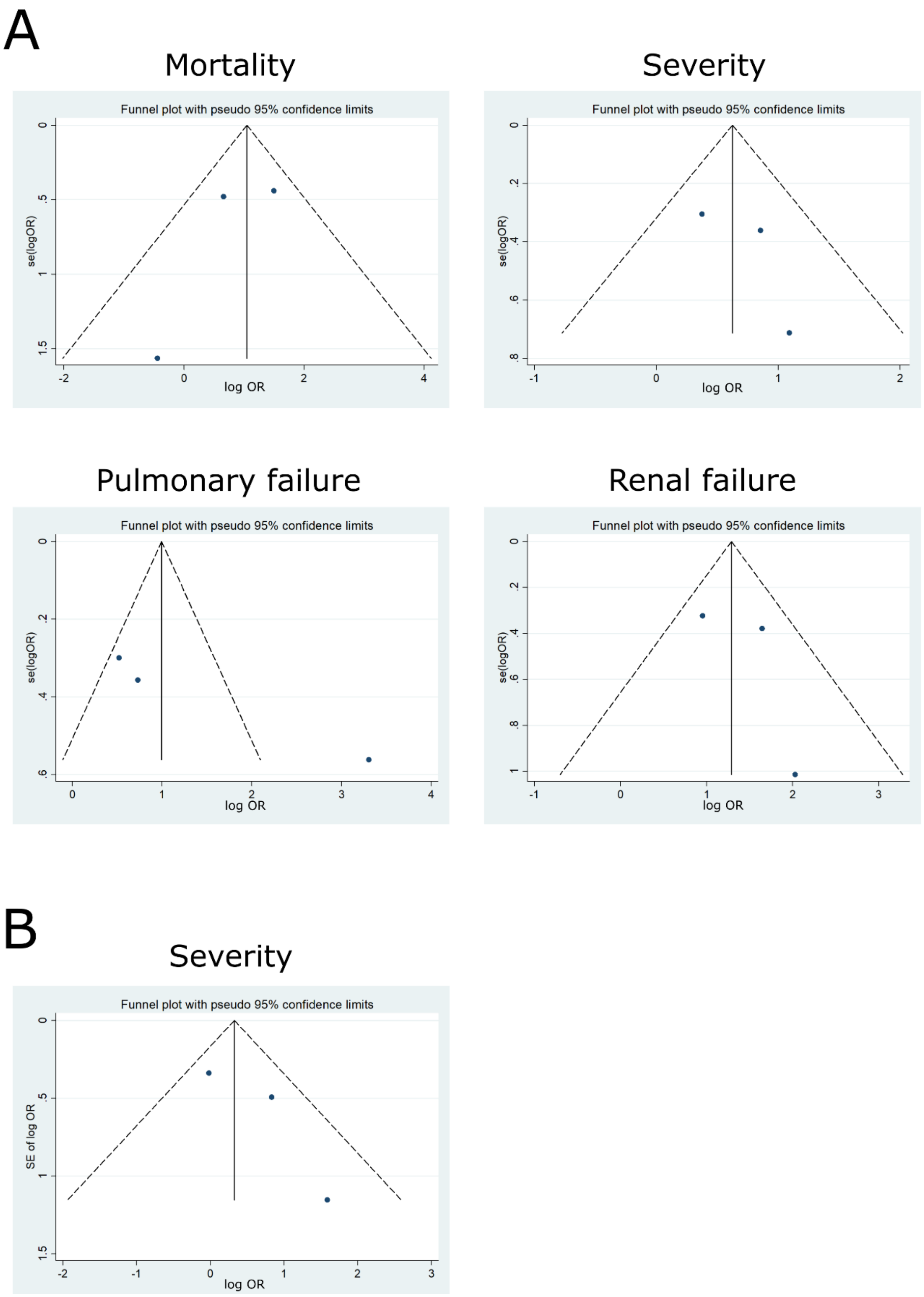

Supplementary Figure 4. Forest plots of mortality, severity, and pulmonary and renal failure related to the meta-analysis where the >5.6 mM (A) and 1.7–5.6 mM (B) seTG vs. <5.6 mM groups were compared (Fig. 6A and 6B, respectively). The two oblique lines mark the pseudo-95% confidence limits.

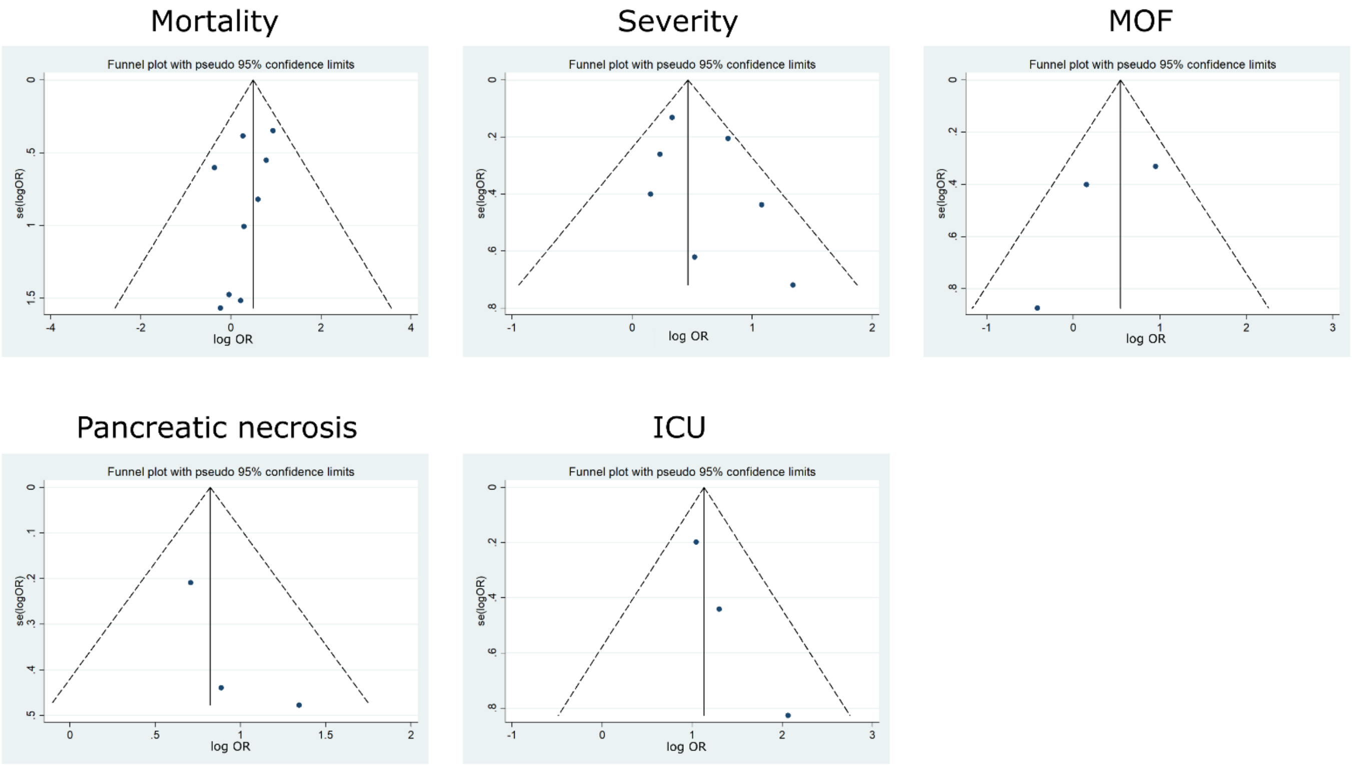

Supplementary Figure 5. Forest plots of mortality, severity, multiple organ failure (MOF), pancreatic necrosis, and intensive care unit admission (ICU) related to the meta-analysis where the >11.3 mM vs. <11.3 mM seTG groups were compared (Fig. 7). The two oblique lines mark the pseudo-95% confidence limits.

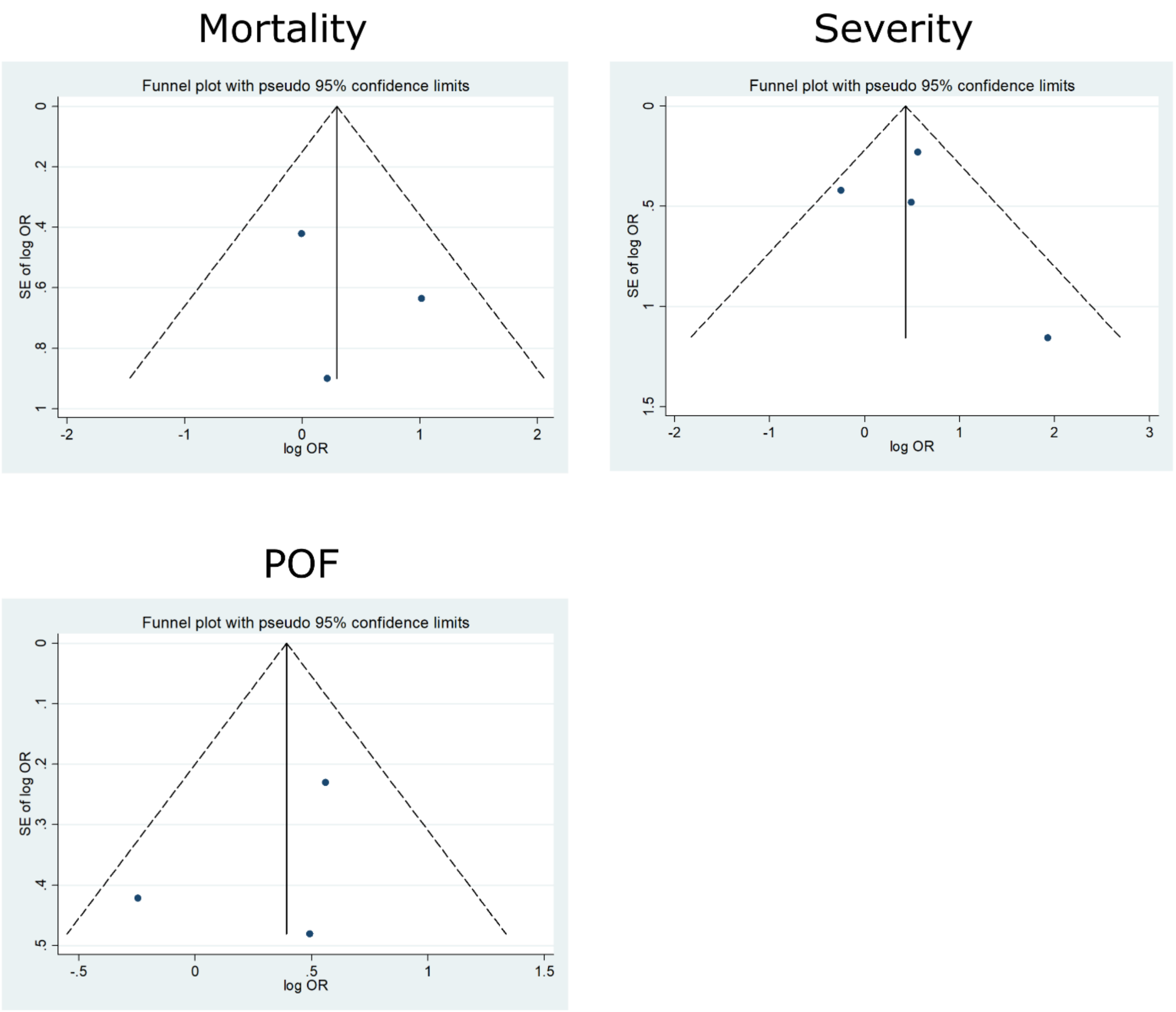

Supplementary Figure 6. Forest plots of mortality, severity and POF related to the meta-analysis where the >11.3 mM vs. 1.7–11.3 mM seTG groups were compared (Fig. 8). The two oblique lines mark the pseudo-95% confidence limits.
